# Antiviral treatment in severe acute hepatitis B

**DOI:** 10.1186/1471-2334-14-S2-P90

**Published:** 2014-05-23

**Authors:** Anca Streinu-Cercel, Oana Streinu-Cercel, Alina Cristina Negut, Marius Stefan, Adrian Streinu-Cercel

**Affiliations:** 1 Department of Infectious Diseases, Carol Davila University of Medicine and Pharmacy, Bucharest, Romania; 2 National Institute for Infectious Diseases, Bucharest, Romania; 3 Polytechnic University of Bucharest, Romania

## Introduction

We aimed to evaluate the clinical, biological and virological impact of antiviral therapy in severe acute hepatitis B. [1]

## Materials and methods

We performed a study in the National Institute for Infectious Diseases “Prof. Dr. Matei Bals”, Bucharest, Romania, randomizing patients between two groups initially: therapy with lamivudine 100 mg/day vs. standard of care (no antiviral therapy) and subsequently between three groups: lamivudine, standard of care and entecavir 0.5 mg/day, when this new analogue became available in Romania.

## Results

HBs antigen to antibody seroconversion was recorded by 24 weeks in 10.1% of the patients in the lamivudine group (p=0.032 compared to control), 42.9% of those in the entecavir group (p=0.053 compared to control), and 22.7% in the control (standard of care) group.

## Conclusions

If antiviral therapy is to be considered in severe acute hepatitis B, entecavir could be regarded as an option, as it might increase the chance of HBs antigen to antibody seroconversion when administered in the first 24 weeks of infection. [2,3] However, further studies are required to ascertain this, and to eventually determine the magnitude of the effect.
